# Recent advances of interfacial modification over tantalum nitride photoanodes for solar water oxidation: a mini review

**DOI:** 10.3389/fchem.2025.1600959

**Published:** 2025-05-09

**Authors:** Pengpeng Wang, Aman Maung Than Oo, Liangji Chen, Doudou Zhang

**Affiliations:** ^1^ Department of Chemistry, Boston College, Merkert Chemistry Center, Chestnut Hill, MA, United States; ^2^ School of Engineering, Macquarie University, Sydney, NSW, Australia

**Keywords:** tantalum nitride, interfacial modification, cocatalysts, hole storage, photoelectrocatalysis, water oxidation

## Abstract

Photoelectrocatalytic (PEC) water splitting represents a highly ideal approach for the efficient conversion of solar energy into sustainable green hydrogen. Although tantalum nitride (Ta_3_N_5_) has emerged as a promising photoanode material, its performance is far below the theoretical limit. Among several photoelectrode design strategies, interfacial modification can be beneficial for suppressing interfacial charge recombination and promoting charge transfer process, which is a key focus in recent research. In the review, a brief overview of recent advances in interfacial modification strategies for Ta_3_N_5_ photoanodes and their influence on the structure-performance relationship are summarized, aiming at an in-depth understanding of the charge-transfer mechanism during PEC water oxidation, and providing insights into designing efficient and stable Ta_3_N_5_ photoanodes for solar-to-fuel conversion through photoelectrocatalysis.

## 1 Introduction

Photoelectrocatalytic (PEC) water splitting has emerged as an exceptionally promising approach for solar-to-fuel conversion, utilizing water as the sole feedstock to generate hydrogen through an environmentally benign and sustainable process that produces zero carbon emissions (Kim et al., 2019; Yu et al., 2021; Nishiyama et al., 2021). In contrast to photocatalytic powder systems, the PEC approach demonstrates enhanced charge separation efficiency, as the combination of irradiation and an external bias facilitates the directional separation and migration of photogenerated charge carriers (He et al., 2019). Moreover, the photovoltage produced under illumination in PEC system can compensate for the applied bias, thereby decreasing energy input requirements in electrocatalysis (EC) system. In addition, the reliance on cost-effective inorganic semiconductors gives PEC a significant economic advantage over hybrid Photovoltaic (PV)-EC approaches (Kim et al., 2019). Because of the complex four-hole process for evolving one oxygen molecule, PEC water oxidation is well recognized as the rate-determining step, therefore, efforts on the photoanode design are of significance (Ye et al., 2019; Seo et al., 2018; Minggu et al., 2010; Li Z. et al., 2013). However, most of the photoanode materials exhibit relatively low performance, far from their theoretical solar-to-hydrogen (STH) efficiencies, primarily due to the low charge separation ability and sluggish charge transport kinetics (Feng et al., 2020; Ning et al., 2019). Most importantly, serious charge recombination issues at the interfaces must be addressed to promote charge transfer kinetics for achieving efficient PEC systems (Haider et al., 2020; Zachaus et al., 2017).

**FIGURE 1:**
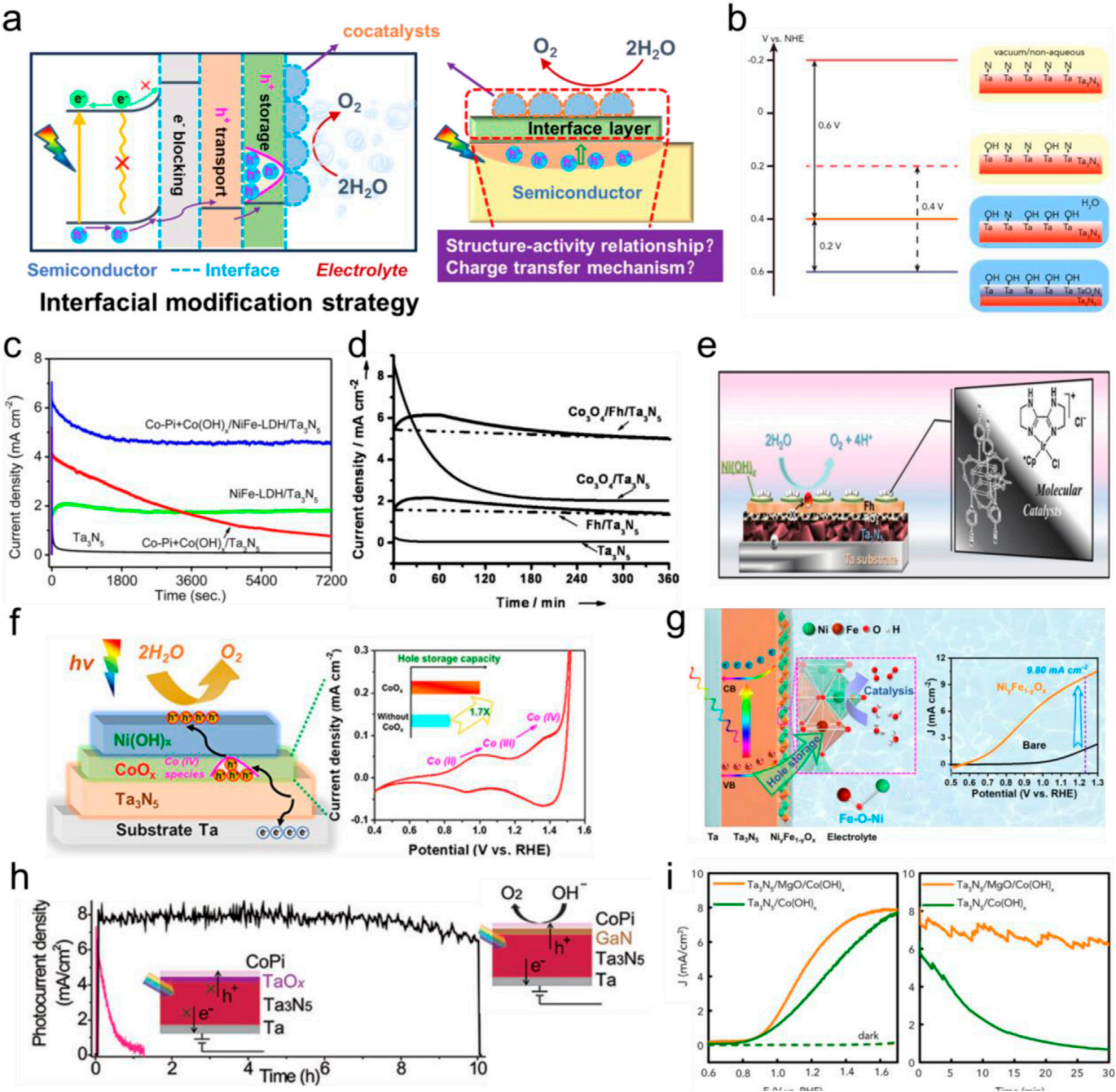
**(a)** The diagram of interfacial modification strategy and the related charge-transfer mechanism study. **(b)** The evolution of Ta_3_N_5_ surface energetics. Reprinted with permission from He et al. (2016). Copyright 2016, Elsevier Inc. **(c)** Steady-state photocurrent density versus time curves of Ta_3_N_5_ photoanodes with different cocatalysts loading. Reprinted with permission from Ref. (Wang L. et al., 2015). Copyright 2015, American chemical society. **(d)** Chronoamperometry measurements of the Ta_3_N_5_ photoanodes with decoration of Fh and Co_3_O_4_ at 1.23 V vs. RHE. Reprinted with permission from Liu et al. (2014). Copyright 2014, Wiley. **(e)** The schematic presentation of the integrated Ta_3_N_5_ photoanode system. Reprinted with permission from Liu et al. (2016). Copyright 2016, Royal society of chemistry. **(f)** Brief schematic diagram of interfacial energetics for the Ni(OH)_x_/CoO_x_/Ta_3_N_5_ photoanode in terms of PEC water oxidation. Reprinted with permission from Wang et al. (2021a). Copyright 2021, American Chemical Society. **(g)** Schematic illustrations of the charge transfer process and PEC water oxidation reaction for Ni_y_Fe_1-y_O_x_/Ta_3_N_5_ photoanodes and photocurrent density-potential curves. Reprinted with permission from Wang et al. (2023). Copyright 2023, American chemical society. **(h)** The stability measurement for the CoPi/GaN/Ta_3_N_5_ and CoPi/Ta_3_N_5_ photoanodes. Reprinted with permission from Zhong et al. (2017). Copyright 2017, Wiley. **(i)** Current-potential and stability curves of Co(OH)_x_/Ta_3_N_5_ photoanodes with and without MgO as a protection layer. Reprinted with permission from He et al. (2016). Copyright 2016, Elsevier Inc.

A wide range of n-type semiconductor materials have been studied in the advancement of PEC water oxidation reaction (Wang S. et al., 2019). Titanium dioxide (TiO_2_) is the earliest material for PEC studies, however, it can only be activated by a small amount of ultraviolet light (<5%) due to its wide gap (3.0–3.2 eV), resulting in the limited theoretical STH efficiency within 1.5%–2.2% (Cho et al., 2013). As for tungsten trioxide (WO_3_), the sluggish charge transport kinetics can lead to serious hole-electron recombination, the low conduction band position can result in low photovoltage (Kim et al., 2013). Although bismuth vanadate (BiVO_4_) is a promising semiconductor photoanode to deliver photocurrent density of 5.8 mA cm^-2^ and applied bias photon-to-current efficiency (ABPE) of 2.7%, its charge transfer kinetics can be limited by short electron diffusion distance and its theoretical STH efficiency can be lower below 10% (9.1%) (Kim and Lee, 2019). Moreover, the low light absorption coefficient (10^3^ cm^-2^), short hole diffusion length can result in poor STH efficiency (<1%) of hematite (ɑ-Fe_2_O_3_) despite its high theoretical STH efficiency (15.9%) (Wheeler et al., 2012). Tantalum nitride (Ta_3_N_5_) is a typical n-type semiconductor with a band gap of 2.1 eV and exhibits broad spectral absorption up to 590 nm (Seo et al., 2018). Moreover, its conduction and valence band positions straddle water reduction and oxidation potentials, respectively, of which this alignment fulfills fundamental thermodynamical requirements necessary for overall solar water splitting (Seo et al., 2018; Chun et al., 2003; Ishikawa et al., 2004; Yu et al., 2025). In theory, Ta_3_N_5_ can reach a maximum photocurrent density of 12.9 mA cm^-2^ under simulated AM 1.5G sunlight (1 sun), accompanied by a theoretical maximum STH of 15.9% (Seo et al., 2018). Till now, numerous efforts have been made to improve both the activity and durability of Ta_3_N_5_ photoanodes. For example, by using gradient Mg doping for band structure engineering, Li and coworkers constructed a In:GaN/Ta_3_N_5_/Mg:GaN heterojunction photoanode, and achieved an ABPE of nearly 3.5% (Fu et al., 2022). Apart from doping/defect control and heterojunction strategy to improve charge separation efficiency, interfacial modification also plays a crucial role in regulating charge transfer and transport behaviors (Ding et al., 2016; Chen et al., 2020; Xiao et al., 2025). As shown in Figure 1a, it has been demonstrated that controlling several interfaces of the semiconductor-electrolyte configuration, including cocatalysts and interfacial layers (such as hole storage layer), is an alternative to lower the reaction barriers and promote charge transfer. However, it still remains challenging to distinguish the functionalities of multilayers clearly in interfacial modification strategies. Consequently, the summary of these interfacial modification strategies and their effects on the OER performance is necessary and critical.

In this review, we summarize some recent advances in the design of Ta_3_N_5_ photoanodes for solar water oxidation through interfacial modification strategies. Then we uncover their charge transfer mechanisms based on an analysis of structure-activity relationship, which may open new avenues for the rational design of a highly efficient photoanode system.

## 2 Interfacial modification strategies on Ta_3_N_5_ photoanodes

### 2.1 Surface study of Ta_3_N_5_

Although Ta_3_N_5_ is a promising candidate for PEC water splitting, it suffers from severe photocorrosion, hindering it from practical applications (Ishikawa et al., 2004). Therefore, investigating the surface structure is helpful for comprehending the surface properties of Ta_3_N_5_ prior to discussing of interfacial modification. Li et al. observed a Ta_3-x_N_5-y_O_y_ layer on the Ta_3_N_5_ surface, and found that its facile removal remarkably reduced charge recombination, resulting in an increased photocurrent (Li M. et al., 2013). Similarly, Nurlaela et al. found that the energetics at the Ta_3_N_5_/H_2_O interface were affected by the surface properties, while the introduction of metallic TaN can result in Fermi level pinning and the subsequent activity decrease (Nurlaela et al., 2014). When Liu et al. fabricated Ta_3_N_5_ electrode from NaTaO_3_ precursor, they found that mixed phases of TaN and TaO led to severe charge recombination (Liu et al., 2016). With regard to this, the introduction of mixed Ar and O_2_ during cooling stage of nitridation process can form a passivation layer. He et al. investigated the energetics of Ta_3_N_5_|H_2_O interface before and after the OER test, and found that the adsorption of H_2_O or hydroxyl species could induce positive shifts of band edge positions, which can explain the high turn-on potential (*V*_*on*_) and lower photovoltage (Figure 1b). (He et al., 2016) In addition, Chen et al. revealed that hydrophobic Ta_3_N_5_ surface after nitridation is not beneficial for water splitting, while impregnating the Ta_2_O_5_ powders in MgSO_4_ solutions could result in hydrophilic surface and more uniform deposition of CoO_x_ as OER catalysts, while Mg doping was found to reduce surface defect densities, contributing to better charge separation efficiency (Chen et al., 2015). Moreover, some theoretical studies for Ta_3_N_5_/H_2_O were also conducted to get insights of the interfacial effects on performance. For instance, Watanabe et al. used density functional theory (DFT) to reveal a downward shift of overall band edge positions once the intermediates were absorbed on the surface of Ta_3_N_5_, a partial Fermi level pinning was observed due to charge redistribution (induced by surface states or absorbed species) (Watanabe et al., 2017). Fan et al. presented a theoretical study of Ta_3_N_5_ photoanodes, and found positive shift of band edge positions by 0.42 V when Ta_3_N_5_ was exposed to H_2_O, while the total shift can be reached 0.85 V once water dissociation occurred on the Ta_3_N_5_ surface (Fan et al., 2019).

These results demonstrate that the direct contact between Ta_3_N_5_ and electrolyte may be harmful for reaction activity, primarily due to surface oxidation and adverse interfacial energetics. Consequently, developing interfacial modification strategies is crucial for minimizing the surface oxidation and improving the performance of Ta_3_N_5_ photoanodes. Table 1 provides a summary of various modification methods (such as cocatalysts, hole-storage layers, physical protection layers) that have been employed to improve the performance of Ta_3_N_5_ photoanodes.

**TABLE 1:** Some representative interfacial modification materials for enhancing the performance of Ta_3_N_5_ photoanodes.

Interfacial layers	Strategies	J@mA cm^-2^ (at 1.23 V_RHE_)	Onset potential (V_RHE_)	Stability	ABPE	Ref
CoPi/Ba-Ta_3_N_5_	cocatalysts	6.7	0.65	20 min, 0.9 V	1.5%	39
FeCoNi-MMO/Ta_3_N_5_	cocatalysts	∼3.8	0.77	60 min, 1.23 V	unknown	40
CoPi + Co(OH)_x_/NiFe-LDH/Ta_3_N_5_	cocatalysts	6.3	0.7	2 h, 1.23 V	unknown	41
IrO_x_/Ta_3_N_5_ nanorods	cocatalysts	3.8	unknown	<20 min, 1.23 V	unknown	43
FeNiO_x_/Ta_3_N_5_-NR	cocatalysts	9.95 (1.05 V)	0.57	70 min, 1.1 V	2.72%	37
NiFeO_x_/Ta_3_N_5_/GaN/Al_2_O_3_	cocatalysts	7.4	0.6	90 min, 1.23 V	1.35%	44
NiCoFe-Bi/In:GaN/Ta_3_N_5_/Mg:GaN	cocatalysts, heterojunction	9.3	0.38	160 min, 1.0 V	3.46%	21
NiCoFe-Bi/Mg: Ta_3_N_5_	cocatalysts, gradient Mg doping	8.5	0.4	5 h, 1.0 V	3.25%	45
CoPi/Co(OH)_x_/Ta_3_N_5_	cocatalysts	6	unknown	150 min, 1.23 V	unknown	46
NiFe-LDH/Ta_3_N_5_	cocatalysts	6–6.7	0.7–0.8	60 min, 1.23 V	unknown	50
NiFe/Ta_3_N_5_	cocatalysts	11.2	0.3	unknown	1.46%	52
Co_3_O_4_/Fh/Ta_3_N_5_	HSL + cocatalysts	5.2	∼0.65	6 h, 1.23 V	unknown	47
Ni(OH)_x_/MoO_3_/Ta_3_N_5_	HSL	<1.5	0.25	24 h, 1.23 V	unknown	48
Ir-Co-complex/Ni(OH)_x_/Fh/TiO_x_/Ta_3_N_5_	HSL + cocatalysts + physical protection layers	12.1	unknown	unknown	2.5%	27
Ni(OH)_x_/CoO_x_/Ta_3_N_5_	HSL	3.2	∼0.6	30 h, 1.23 V	unknown	49
Ni_y_Fe_1-y_O_x_/Ta_3_N_5_	HSL or cocatalysts	9.8	∼0.6	3 h, 1.23 V	1.66%	51
CoPi/GaN/Ta_3_N_5_	Protection layers + cocatalysts	8 (1.2 V)	0.65	10 h, 1.2 V	1.5%	53
Co(OH)_x_/MgO/Ta_3_N_5_	Protection layers + cocatalysts	<6	∼0.8	30 min, 1.23 V	unknown	28
Co(OH)_x_/Ta_3_N_5_/NbN_x_	Protection layers + cocatalysts	3.5	unknown	140 min, 1.23 V	unknown	55
CoPi/Ta_3_N_5_/Ta_2_N/Ta	Protection layers + cocatalysts	8.1	unknown	70 min, 1.23 V	unknown	56

### 2.2 Cocatalysts

A wide range of OER catalysts have been applied for improving the activity of photoanodes, including IrO_x_ (Wang Z. et al., 2015), CoPi (Wang L. et al., 2019; Wang et al., 2022), CoO_x_ (Lee et al., 2019; Liao et al., 2012), and Fe-Co-Ni based (oxy-)hydroxides (Pihosh et al., 2020; Zhang et al., 2022). In general, the decoration of cocatalysts on the surface of photoanodes were proved to be significantly reduce the OER energy barrier, allowing photogenerated holes to be kinetically favored toward OER. Meanwhile, it has been reported that cocatalysts loading can efficiently passivate surface states and suppress interfacial recombination centers as well as provide more active sites to enhance the photocurrent densities (Yu et al., 2025; Liao et al., 2012). In general, the strategy is always combined with doping method to enhance performance, in some cases multiple layers of cocatalysts are also applied. For instance, Li et al. found that Ba doping could suppress the formation of less conductive Ta_5_N_6_ phase within Ta_3_N_5_ photoanode and shift flat-band potential negatively. With the addition of CoPi as the cocatalysts, a photocurrent density of 6.7 mA cm^-2^ was reached at 1.23 V (Li et al., 2013c). Haleem et al. synthesized FeNiCoO_x_ as the cocatalysts on Ta_3_N_5_ via photo-assisted electrodeposition and exhibited stable photocurrent densities around 4 mA cm^-2^ at 1.23 V for 2 h (Abdel Haleem et al., 2017). Wang et al. obtained a solar photocurrent of 6.3 mA cm^-2^ at 1.23 V for Ta_3_N_5_ nanorod photoanode by the sequential modifications of NiFe-layered double hydroxide (LDH), Co(OH)_x_ and CoPi as the cocatalysts (Wang L. et al., 2015). Moreover, a steady photocurrent of ∼5 mA cm^-2^ was remained at 1.23 V for at least 2 h of irradiation (Figure 1c). Despite being as an efficient OER catalysts, IrO_x_ modified Ta_3_N_5_ photoanodes have been displayed to exhibit rapid decay of OER performance (Yokoyama et al., 2011; Li et al., 2013d). This is primarily due to incomplete coverage of IrO_x_ on Ta_3_N_5_ interface and the subsequent Ta_3_N_5_ surface oxidation, where Fermi level pinning could be formed to undermine the charge separation efficiency. Pihosh et al. fabricated highly-conductive polycrystalline Ta_3_N_5_-nanorods for advantageous light harvesting, FeNiO_x_ cocatalyst loading can result in efficient charge separation and a completely saturated photocurrent density of 9.95 mA cm^-2^ at 1.05 V (Pihosh et al., 2020). Later, the same group deposited an ultrathin NiFeO_x_ electrocatalyst layer on semitransparent Ta_3_N_5_ photoanode and dramatically improved the stability and generated a photocurrent density of 7.4 mA cm^-2^ at 1.23 V (Higashi et al., 2022). Furthermore, this photoanode was coupled with CuInSe_2_ to form PEC-PV tandem cells and achieved an initial STH efficiency of 9%. Moreover, combined with band structure engineering of Ta_3_N_5_ photoanodes or heterojunction construction, the decoration of NiCoFe-Bi cocatalysts can deliver exceptional ABPE values toward solar water oxidation (Fu et al., 2022; Xiao et al., 2020). He et al. deposited CoPi and Co(OH)_2_ nanosheet cocatalysts on the Ta_3_N_5_ surface and the PEC performance was found to be dramatically enhanced (He et al., 2017). Systematical studies indicated that light-induced O^·^ radicals could lead to the formation of Ta-O-Co bonds between Co(OH)_2_ and Ta_3_N_5_, thereby largely suppressing Fermi-level pinning, reducing charge recombination and accelerating charge transfer efficiency.

Nevertheless, it is significant to recognize that both the kinetics and energetics at the electrode/electrolyte surface can be regulated upon cocatalysts loading, as the consequence of the decrease of charge extraction barrier. Moreover, sometimes the cocatalyst decoration can also passivate surface defects and decrease the possibility of charge recombination. Therefore, the charge transfer process can be promoted, both the photocurrent density and durability can be improved.

### 2.3 Hole-storage layers

The concept of “hole-storage-layer” (HSL) was first proposed by Liu et al. when they used ferrihydrite (Fh) to protect Ta_3_N_5_ from photocorrosion (Liu et al., 2014). As displayed in Figure 1d, the decoration of Fh could promptly extract and store photogenerated holes from Ta_3_N_5_, thereby prolonging OER durability (at 1.23 V) for 6 h without obvious photocurrent degradation. Although Co_3_O_4_ nanoparticles cocatalysts impressively accelerate charge transfer kinetics and improve activity, Ta_3_N_5_ was not effectively protected from photocorrosion, highlighting the vital role of HSL in electrode design. Later, Ni(OH)_x_/MoO_3_ bilayer was designed as HSL for remaining the photocurrent density stable over 24 h, benefiting from the high hole mobility and efficient hole extraction contributed from this HSL (Liu et al., 2015). The most impressive demonstration for HSL was reported by Liu et al., in 2016, they prepared an integrated Ta_3_N_5_ photoanode for delivering a photocurrent density of 12.1 mA cm^-2^ (approaching the theoretical value of 12.9 mA cm^-2^) and an ABPE of 2.5%, where the Ni(OH)_x_ and Fh were combined as HSLs, the surface of Ta_3_N_5_ was passivated via Ar/O_2_ gas, along with TiO_x_ as an electron blocking layer and Co-Ir molecular complexes as the OER cocatalysts (Figure 1e). (Liu et al., 2016) Intensity-modulated photocurrent spectroscopy (IMPS) was utilized for revealing the role of HSL, showing that the modification of HSLs only suppressed interfacial charge recombination but not influenced the charge transfer kinetics. Recently, Wang et al. used Ni(OH)_x_/CoO_x_ as the HSL for improving photostability of Ta_3_N_5_ for 30 h, while the reversible formation of Co(IV) species within the ultrathin CoO_x_ layer was considered to be the main cause for facilitating hole extraction toward Ni(OH)_x_, as illustrated in Figure 1f (Wang et al., 2021a).

It is worth noting that cocatalysts and HSLs often exhibit synergistic effects, as demonstrated by Ni-Fe oxide/hydroxides decorated Ta_3_N_5_ photoanodes. By using electrodeposition method, Fang et al. prepared NiFe-LDH and Ni_0.9_Fe_0.1_OOH as cocatalysts, dramatically improving the PEC performance of Ta_3_N_5_ photoanodes (Fang et al., 2018). Domen et al. deposited FeNiO_x_ cocatalysts on Ta_3_N_5_ nanorod photoanodes and achieved an exceptional photocurrent density of 9.95 mA cm^-2^ at 1.05 V, accompanied with ABPE of 2.72% (Pihosh et al., 2020). Despite these advancements, the charge transfer mechanism of Ni-FeO_x_/Ta_3_N_5_ photoanodes for PEC water oxidation remains unclear. With this regard, Wang et al. prepared Ni-Fe oxyhydroxides (Ni_y_Fe_1-y_O_x_) with different Ni and Fe loading ratios for modifying Ta_3_N_5_ photoanodes and elucidated the reaction mechanism (Wang et al., 2023). As shown in Figure 1g, it was found that a photocurrent density of 9.8 mA cm^-2^ was reached at 1.23 V upon loading Ni_0.5_Fe_0.5_O_x_, while the ABPE value could be increased from 0.1% to 1.66%. They revealed the role of Ni_y_Fe_1-y_O_x_ as both the cocatalysts and HSL. Ni species primarily store holes from Ta_3_N_5_, while the Fe-O-Ni species are responsible for promptly extracting and transferring the stored holes to electrode/electrolyte interface, the synergetic effects can result in effective charge transfer efficiencies for OER. Similarly, Dong et al. revealed that Fe catalysts within NiFe-catalysts effectively promoted charge separation and hole transfer of Ta_3_N_5_ photoanode, while Ni catalyst nanolayers provided catalytic active-sites for PEC water splitting (Dong et al., 2023).

After the full comprehension, it is hard to distinguish cocatalysts from HSLs in photoanode modification over solar water oxidation. Both the cocatalysts and HSLs can facilitate charge extraction, separation, transport and injection, except that cocatalysts seem to demonstrate more obvious advantages over HSLs in charge injection at electrode/electrolyte interface. On the other hand, the decoration of HSLs can generally protect Ta_3_N_5_ from photocorrosion and improve the stability, while sometimes cocatalyst nanoparticles can’t be covered completely on Ta_3_N_5_ surface to get rid of photoanode degradation during long-term water splitting operation, although photocurrent density can be enhanced. Taking Fe-Co-Ni based (oxy-)hydroxide cocatalysts as the example, there are several complex steps including charge extraction, hole-storage processes (in the form of high-valence species) and hole-injection/release for water oxidation. Overall, from the aspect of interfacial modification, it must be more significant to concentrate on energy diagram matching and material choices for rationally designing highly efficient photoanode system.

### 2.4 Physical protection layers

Apart from cocatalysts and HSLs, transparent oxide/nitride films were widely used as the protection layers for enhancing the stability of photoelectrodes. Moreover, the activity could be also boosted with the assistence of cocatalysts. For example, Zhong et al. grew a ∼50 nm GaN layer on Ta_3_N_5_ by nitriding the deposited GaO_x_, with the further loading of CoPi cocatalyst, the GaN protected Ta_3_N_5_ photoanode exhibited stable photocurrent densities over 8 mA cm^-2^ for 10 h (Figure 1h). (Zhong et al., 2017) By comparison, it was found that the CoPi/Ta_3_N_5_ photoanode showed a negligible photocurrent density within 1 h, emphasizing the critical role of GaN in preventing the penetration of electrolytes into the Ta_3_N_5_ surface. Zhang et al. found that the deposited TiO_2_ on the Ta_3_N_5_ surface significantly removed surface states, reduced charge recombination and promoted charge separation, the onset potential was hence shifted negatively (Zhang et al., 2016). When He et al. introduced a compact MgO layer between Ta_3_N_5_ and outmost Co(OH)_x_ cocatalyst via atom layer deposition (ALD) method, they found that MgO could not only effectively separate Ta_3_N_5_ from electrolytes or active oxygen species, but also improve the attachment of cocatalysts on Ta_3_N_5_ surface (He et al., 2016). Therefore, the fill factor of J-V curve for Ta_3_N_5_ photoanode was improved, implying the promotion of charge separation. Moreover, the stability of PEC water oxidation system was obviously enhanced (Figure 1i). Considering the high Schottky barriers formed between Ta_3_N_5_ film and Ta substrate to hinder electron transfer, Wang et al. deposited several different back contact layers such as NbN_x_, TiN_x_ and CdS and found that the Ta_3_N_5_/NbN_x_/Ta photoanode yielded highest photocurrent density, demonstrating the effective promotion of electron transfer from Ta_3_N_5_ to the substrate (Wang et al., 2016). The same group later found that the presence of Ta_2_N interlayer can facilitate electron transfer between Ta_3_N_5_ and Ta substrate, thereby delivering an improved photocurrent density of 8.1 mA cm^-2^ at 1.23 V with the additional assistance of CoPi cocatalysts (Nurlaela et al., 2020).

It should be mentioned that both the decoration of cocatalysts and HSLs can be employed to provide additional driving force for charge separation and extraction within the photoanodes over solar water oxidation, while the primary functionality of the physical protection layers focuses on the inhibition of electron-hole recombination, promotion of electron transfer to substrate as well as the interfacial passivation effects.

## 3 Conclusion and outlook

**FIGURE 2:**
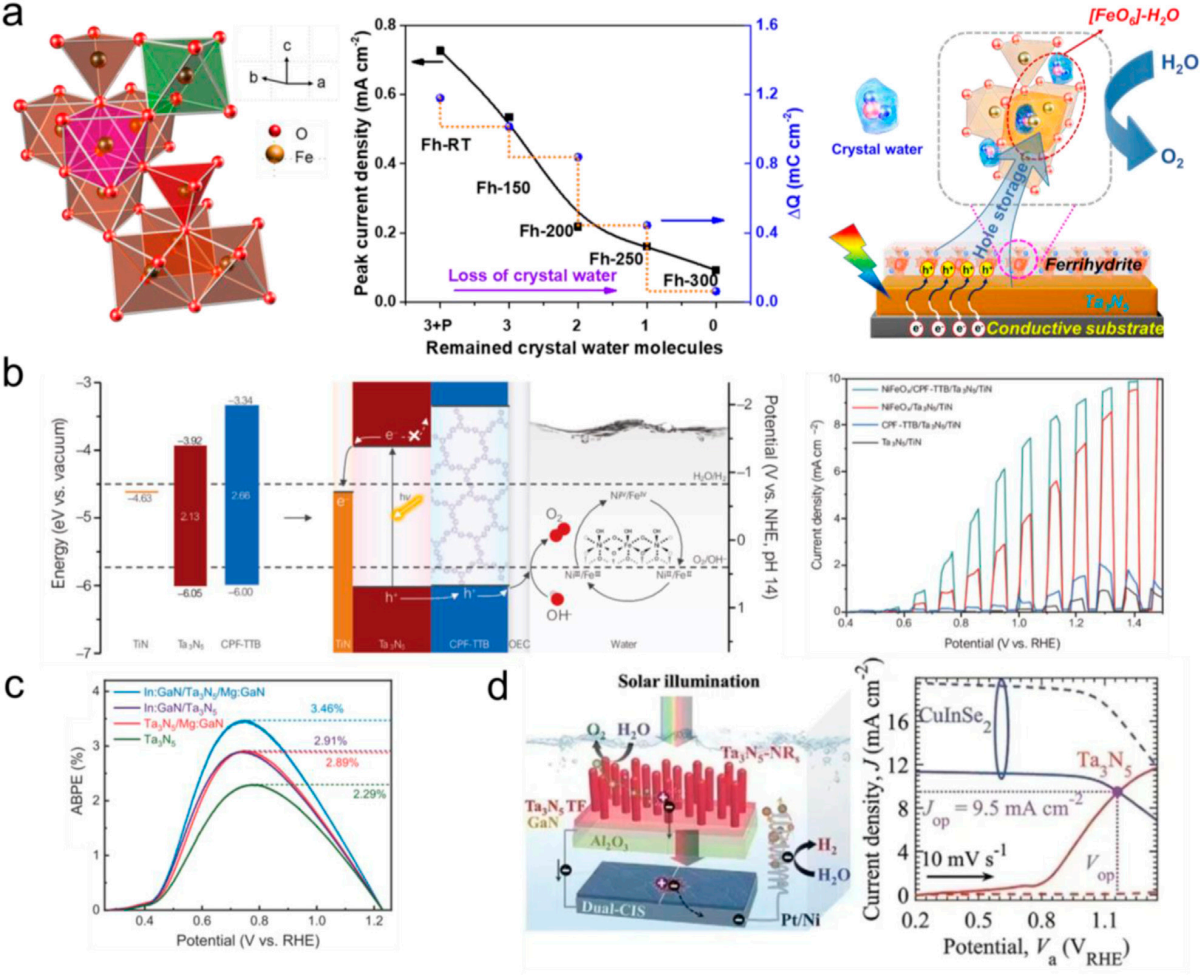
**(a)** Basic structural motif of Fh, PEC water oxidation performance changes of Fh/Ta_3_N_5_ photoanodes with sequential loss of crystal water within Fh, and the corresponding diagram of charge-transfer and hole-storage active sites. Reprinted with permission from Wang et al. (2021b). Copyright 2021, Wiley. **(b)** Energy band diagrams energy of TiN, Ta_3_N_5_, and CPF-TTB and schematic illustration of the NiFeO_x_/CPF-TTB/Ta_3_N_5_/TiN photoanode during PEC water oxidation, and photocurrent density-potential curves of the above photoanodes. Reprinted with permission from Yang et al. (2024). Copyright 2024, Wiley. **(c)** ABPE curves of the Ta_3_N_5_-based photoanodes, with NiCoFe-Bi as the cocatalysts. Reprinted with permission from Fu et al. (2022). Copyright 2022, Springer Nature. **(d)** Schematic diagram and working principle of tandem device comprised of serially connected semi-transparent Ta_3_N_5_ photoanode with dual-CuInSe_2_ photovoltaic cells and Pt/Ni electrode, and the J-V curves of dual-CuInSe_2_ cells and Ta_3_N_5_ photoanode. Reprinted with permission from Pihosh et al. (2023). Copyright 2023, Wiley.

As mentioned above, great efforts have been made to develop efficient and stable Ta_3_N_5_ photoanodes by interfacial modification strategies such as cocatalysts loading, HSL decoration and the introduction of physical protection layer. It is worth noting that these methods have been always combined through rational interfacial modification engineering to promote charge separation, boost reaction kinetics and address the instability issues. Currently, the achievable photocurrent density of Ta_3_N_5_ photoanode reaches 12.1 mA cm^-2^ (Liu et al., 2016), very close to the theoretical value of 12.9 mA cm^-2^. However, the onset potential is 0.38 V, far from the theoretical value (<0 V), and the recorded ABPE reported is only 3.46% (Fu et al., 2022), posing a significant bottleneck for the practical application of Ta_3_N_5_ photoelectrode material. Therefore, although interfacial modification strategies have been developed for many years, several challengs still remains unresolved. (i) The structure properties of most of the interfacial modification layers lack in-depth research and charge transfer pathway under operando condition still remain unclear. (ii) There is a necessity to develop more interfacial materials with superior charge mobility properties and suitable energy positions. (iii) Defects and the internal electric field of Ta_3_N_5_ material could be further optimized to secure a more efficient charge separation efficiency. (iv) More in-depth studies of synergetic effects between interfacial modification and other techniques are required to design improved structures and systems. Accordingly, we will conduct the discussion as follows:

First, although numerous interfacial layers have been developed to significantly enhance the performance of Ta_3_N_5_ photoanode, few researches have focused on the effect of the phase/structure of interfacial layers on OER activity. For instance, by carefully regulating the phase structures of FeOOH, it was found that highly crystalline β-FeOOH and mixed-phase FeOOH(α+β) cocatalysts could achieve higher photocurrent densities for BiVO_4_ photoanodes than those of amorphous FeOOH and single phase FeOOH, respectively, primarily due to optimized crystalline structure and abundant oxygen vacancies (Zhang et al., 2018; Wang et al., 2025). By dehydrating the Fh via a careful calculation, the gradual weakening of the hole-storage capability of Fh/Ta_3_N_5_ photoanodes was observed, primarily caused by the irreversible loss of crystal water and mutation of coordination symmetry of [FeO_6_] hydration units (Wang et al., 2021b). As a consequence, the hydration structure of Fh was identified to be responsible for hole-storage ability of Fh/Ta_3_N_5_ photoanodes (Figure 2a). Moreover, the application of some (quasi-)operando spectroscopies (such as FT-IR and Raman) are recommended to detect interfacial structure evolutions during PEC water oxidation for revealing deeper insights into the charge transfer mechanism (Pan et al., 2023; Liu et al., 2025). In a word, the careful regulation of interfacial structures of the interfacial layers and the in-depth understanding of charge transfer pathway will be beneficial for the rational design of photoanodes in the near future.

Second, learned from interface energetical engineering strategy in solar cells, designing interfacial layers with high hole mobility/transport properties between cocatalysts and Ta_3_N_5_ photoanode is also critical, of which the work function matching should be also considered. Recently, Yang et al. elaborately introduced a conjugated polythiophene framework (CPF-TTB) as the hole-selective layer between Ta_3_N_5_ and the outmost NiFeO_x_ cocatalyst, the enhanced hole extraction enabled the NiFeO_x_/CPF-TTB/Ta_3_N_5_/TiN photoanode to generate a remarkable photocurrent density of 9.12 mA cm^-2^ for water oxidation at 1.23 V (Figure 2b). (Yang et al., 2024) Moreover, the energy band diagrams of substrate TiN, Ta_3_N_5_ and CPF-TTB were determined to be advantageous for efficient charge extraction from Ta_3_N_5_. Specially, the presence of CPT-TTB could restrain charge recombination and expedite hole transport from Ta_3_N_5_ to NiFeO_x_ by the formation of energetically favorable type II heterojunction. This example provides a novel perspective in the design of interfacial layer by considering energy band diagram, and hole transport through organic-inorganic hybrid method.

Third, the rational design of highly efficient Ta_3_N_5_ photoanode requires not only the interfacial layer with excellent activity and highly matched energetics between multiple layers, but also the optimized Ta_3_N_5_ electrode with effective charge separation and transport properties. Xiao et al. used gradient Mg doping in Ta_3_N_5_ to induce a gradient of band edge energetics for greatly enhancing charge separation efficiency. In addition, defect-related recombination could be significantly suppressed due to the passivation effect of Mg dopants on deep-level defects. As a consequence, the Mg-doped Ta_3_N_5_ photoanode delivered a low onset potential of 0.4 V with the assistence of Ni-Co-Fe-Bi cocatalyst, accompanied with ABPE of 3.25% (Xiao et al., 2020). Later, the same group designed the In:GaN/Ta_3_N_5_/Mg:GaN heterojunction photoanode and achieved a record ABPE of 3.46% for Ta_3_N_5_-based photoanodes(Figure 2c), this excellent performance was attributed to the enhanced bulk carrier separation capability and better injection efficiency at the photoanode/electrolyte interface (Fu et al., 2022). These results highlight the effectiveness of proper interface engineering for achieving an efficient PEC water splitting system.

Finally, achieving the unassisted PEC water splitting with sunlight as the sole energy input and no external bias, is the ultimate goal for sustainable solar-to-hydrogen energy conversion. However, it remains challenging to achieve this goal using a single Ta_3_N_5_ photoanode. A more practical approach involves constructing tandem cells, either combining a Ta_3_N_5_ photoanode with a photocathode or integrating it with a photovoltaic(PV) cell (Higashi et al., 2022; Higashi et al., 2019; Pihosh et al., 2023). Domen and coworkers prepared transparent Ta_3_N_5_-NRs/Ta_3_N_5_-TF/GaN/Al_2_O_3_ photoanodes that could deliver the photocurrent density of 10.8 mA cm^-2^ at 1.23 V (Pihosh et al., 2023). Subsequently, these Ta_3_N_5_ photoanodes were connected in series and driven by a dual-CuInSe_2_ solar cell to achieve a matching photocurrent density of 9.5 mA cm^-2^ with an operating voltage of 1.16 V (Figure 2d).

Typically speaking, an efficient interfacial engineering may meet several criteria: (1) Low-defect interfaces of semiconductors and lattice-matched semiconductor/interfacial layers to minimize defects, (2) suitable energy level alignment for ensuring favorable charge transfer, (3) compatible fabrication processes for integrated photoanodes. These considerations can be beneficial for screening the optimized interfacial materials for the rational design of highly efficient water splitting systems. When effectively implemented, the interfacial engineering strategies (discussed above) can efficiently improve the PEC performance of Ta_3_N_5_ photoanodes by addressing a series of challenges associated with the interfacial defects and charge extraction barriers.

Meanwhile, in the past few years, some advanced (quasi-)operando techniques (such as Raman, FT-IR, XAS) have been widely used to grasp charge transfer pathway, in combination with DFT theoretical calculations. For instance, Ismail et al. employed operando XAS to investigate interfacial dynamics at the NiFeOOH/ɑ-Fe_2_O_3_ interface and found that the formation of FeOOH plays a critical role in the surface passivation and hole extraction of ɑ-Fe_2_O_3_ (Ismail et al., 2021). When Pan et al. decorated ZnCoFe polyphthalocyanine on BiVO_4_ to form core-shell photoanode, they found that the interfacial charge transfer can be facilitated by lowering the Fe d band center and orbital spin (Pan et al., 2023). Based on quasi-operando Raman measurements and DFT calculation, they revealed that the promotion of *OOH desorption is the potential limiting step for modulating the catalytic activity. Recently, Liu et al. found that the accumulated high-density holes on ɑ-Fe_2_O_3_ surface could form adjacent Fe^V^ = O intermediates that effectively activate surface-adsorbed H_2_O molecules via the hydrogen-bonding effect, as revealed by operando Raman measurements and *ab initio* molecular dynamics simulations (Liu et al., 2025). Therefore, one potential research technical route may manifest: (1) Carefully regulating phase structure or bonding characteristics of interfacial layers modified photoanodes, (2) investigating the changes of PEC water oxidation activity and charge-transfer kinetics with the structural changes of interfacial layers, (3) exploring the real-time evolution of interfacial layers during PEC water oxidation by using operando techniques and theoretical calculations. This assumption must be one of the research directions regarding in-depth understanding of charge-transfer mechanism.

In addition, the integration of photoanodes with other research fields, such as thermal catalysis and organic synthesis, to advance PEC water splitting. Thermal catalysis is known to rely on elevated temperatures, once combined the photoanodes with thermal catalysts, both solar energy and thermal energy can be utilized to improve water splitting conversion efficiency without external bias. On the other hand, as Ta_3_N_5_ is a promising alternative for overall water splitting, considering that water oxidation half reaction is the rate-determining step, we consider to replace it with organic synthesis to obtain high-value chemicals. Consequently, combining the hydrogen production on the cathode with organic molecule activation on the anode may make a difference. This highlights some extensive study potentials of photoanodes with some hybrid catalytic systems.

In summary, this mini-review provides a comprehensive overview of representative advancements in interfacial modification strategies for Ta_3_N_5_ photoanodes, highlighting the crucial role of interfacial structure engineering in promoting charge separation, transfer and enhancement of PEC performance. We also provide suggestions for achieving good performance through interfacial modifications of Ta_3_N_5_ photoanodes. An in-depth understanding for the physical and chemical properties of interfacial layers and the structure-activity relationship can bring enlightenment for electrode design. High-performance photoanodes can be fabricated through developing interfacial materials with high charge mobility and suitable energetic matching, defect control and gradient doping of Ta_3_N_5_, as well as integrating with PV-PEC coupling systems, etc. We believe that the combination of interfacial modification and other strategies will be effective in achieving a high-performance Ta_3_N_5_ photoanode system for solar-to-fuel conversion and other energy, catalytic applications in the near future.
